# Retinal imaging for the assessment of stroke risk: a systematic review

**DOI:** 10.1007/s00415-023-12171-6

**Published:** 2024-03-02

**Authors:** Zain Girach, Arni Sarian, Cynthia Maldonado-García, Nishant Ravikumar, Panagiotis I. Sergouniotis, Peter M. Rothwell, Alejandro F. Frangi, Thomas H. Julian

**Affiliations:** 1 Sheffield Medical School, University of Sheffield, Beech Hill Rd, Broomhall, Sheffield, UK; 2 Manchester Royal Infirmary, Manchester University NHS Foundation Trust, Oxford Rd, Manchester, UK; 3 Centre for Computational Imaging and Simulation Technologies in Biomedicine, School of Computing, University of Leeds, Leeds, UK; 4 Division of Evolution, Infection and Genomics, School of Biological Sciences, Faculty of Biology, Medicine and Health, University of Manchester, Manchester, UK; 5 European Molecular Biology Laboratory, European Bioinformatics Institute (EMBL-EBI), Wellcome Genome Campus, Hinxton, UK; 6 Manchester Centre for Genomic Medicine, Saint Mary’s Hospital, Manchester University NHS Foundation Trust, Manchester, UK; 7 Manchester Royal Eye Hospital, Manchester University NHS Foundation Trust, Oxford Rd, Manchester, UK; 8 Wolfson Centre for the Prevention of Stroke and Dementia, University of Oxford, Oxford, UK; 9 Division of Informatics, Imaging and Data Sciences, School of Health Sciences, Faculty of Biology, Medicine and Health, University of Manchester, Manchester, UK; 10 School of Computer Science, Faculty of Science and Engineering, University of Manchester, Kilburn Building, Manchester, UK; 11 Christabel Pankhurst Institute, The University of Manchester, Manchester, UK

**Keywords:** Stroke, Artificial intelligence, Prediction, Biomarkers, Retina, Deep learning

## Abstract

**Background:**

Stroke is a leading cause of morbidity and mortality. Retinal imaging allows non-invasive assessment of the microvasculature. Consequently, retinal imaging is a technology which is garnering increasing attention as a means of assessing cardiovascular health and stroke risk.

**Methods:**

A biomedical literature search was performed to identify prospective studies that assess the role of retinal imaging derived biomarkers as indicators of stroke risk.

**Results:**

Twenty-four studies were included in this systematic review. The available evidence suggests that wider retinal venules, lower fractal dimension, increased arteriolar tortuosity, presence of retinopathy, and presence of retinal emboli are associated with increased likelihood of stroke. There is weaker evidence to suggest that narrower arterioles and the presence of individual retinopathy traits such as microaneurysms and arteriovenous nicking indicate increased stroke risk. Our review identified three models utilizing artificial intelligence algorithms for the analysis of retinal images to predict stroke. Two of these focused on fundus photographs, whilst one also utilized optical coherence tomography (OCT) technology images. The constructed models performed similarly to conventional risk scores but did not significantly exceed their performance. Only two studies identified in this review used OCT imaging, despite the higher dimensionality of this data.

**Conclusion:**

Whilst there is strong evidence that retinal imaging features can be used to indicate stroke risk, there is currently no predictive model which significantly outperforms conventional risk scores. To develop clinically useful tools, future research should focus on utilization of deep learning algorithms, validation in external cohorts, and analysis of OCT images.

**Supplementary Information:**

The online version contains supplementary material available at 10.1007/s00415-023-12171-6.

## Introduction

Stroke is the second leading cause of death, and a leading cause of disability. Current estimates suggest that 15 million people suffer from stroke worldwide, with these resulting in 5 million deaths. The two main subtypes of stroke are ischemic (~80% of cases) and hemorrhagic stroke (~20% of cases) [1]. Crucially, it is estimated that up to 80% of strokes are preventable [2]. With that in mind, it is essential that those at risk are identified early so that effective primary prevention strategies can be implemented.

**Fig. 1:**
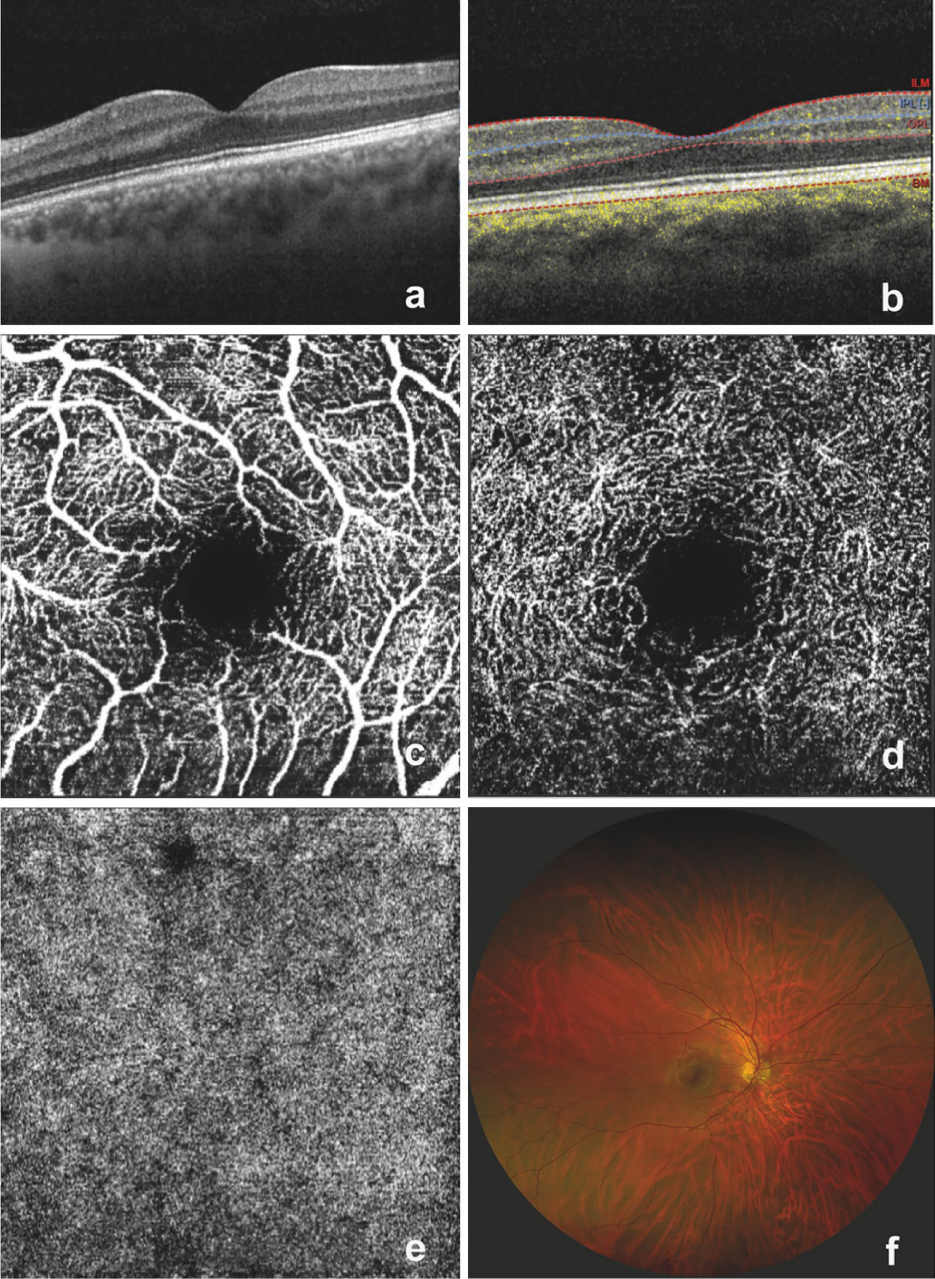
A selection of retinal images which illustrate normal anatomy in optical coherence tomography (OCT), OCT angiography (OCT-A), and scanning laser ophthalmoscopy: **a** a structural OCT image of the macula; **b** a structural OCT with OCT-A flow overlay (highlighted by yellow dots, which indicate blood flow) and with automated segmentation at the internal limiting membrane (ILM), inner plexiform layer (IPL), outer plexiform layer (OPL) and Bruch’s membrane (BM); **c** an en face macula OCT-A image (superficial vascular plexus slab); **d** an en face macula OCT-A image (deep vascular plexus slab); **e** an en face macula OCT-A image (choriocapillaris slab); **f** a wide-field retinal image acquired using scanning laser ophthalmoscopy

The retina is the only tissue that permits direct, non-invasive visualization of the microvasculature and central nervous system. Given the retina’s homology with the brain in terms of anatomy, physiology, and embryology, there is great potential for the retina to play a role in the prediction of cerebrovascular disease [3]. During routine clinical practice in both hospital and community (optometry) settings, optometrists and ophthalmologists can assess the retina in a direct, non-invasive, inexpensive manner through ocular imaging, including scanning laser ophthalmoscopy, fundus photography, optical coherence tomography (OCT), and OCT angiography (OCT-A) (Fig. 1). With OCT, scanning laser ophthalmoscopy, and fundus photography technology constantly developing (and with increasingly widefield imaging available), opportunities to detect clinically useful retinal biomarkers reflecting stroke risk are now abundant. Further, artificial intelligence (AI)-enabled analysis of retinal imaging has been shown to predict stroke with good accuracy using machine learning approaches [4, 5]. AI has also proven capable of inferring a range of cardiovascular disease relevant factors from retinal images including blood pressure, smoking status, sex, and age—inferences impossible for human observers to make [6]. Combining modern imaging technologies with cutting-edge AI systems, the future for stroke prediction from retinal images appears bright.

Whilst there is an extensive evidence synthesis literature base surrounding the role of stroke risk biomarkers including silent brain infarcts [7], proteinuria [8], pulse pressure [9], and carotid atherosclerosis [10], there is currently no systematic review detailing the relationship between retinal biomarkers and future stroke risk. With a view to summarizing the current evidence and opportunities in this domain, in this review, we explore the role of retinal biomarkers as indicators of future stroke risk and discuss the future of research in this area. To this end, this review 1) describes studies featuring AI-enabled methodologies to predict stroke; 2) summarizes the evidence surrounding the role of specific retinal biomarkers in stroke prediction; and 3) provides an assessment of the limitations and opportunities in this domain.

## Methods

### Protocol registration

This review was prospectively registered to PROSPERO [11]. The registration number for this review is CRD42023389223.

### Literature search strategy

**Fig. 2:**
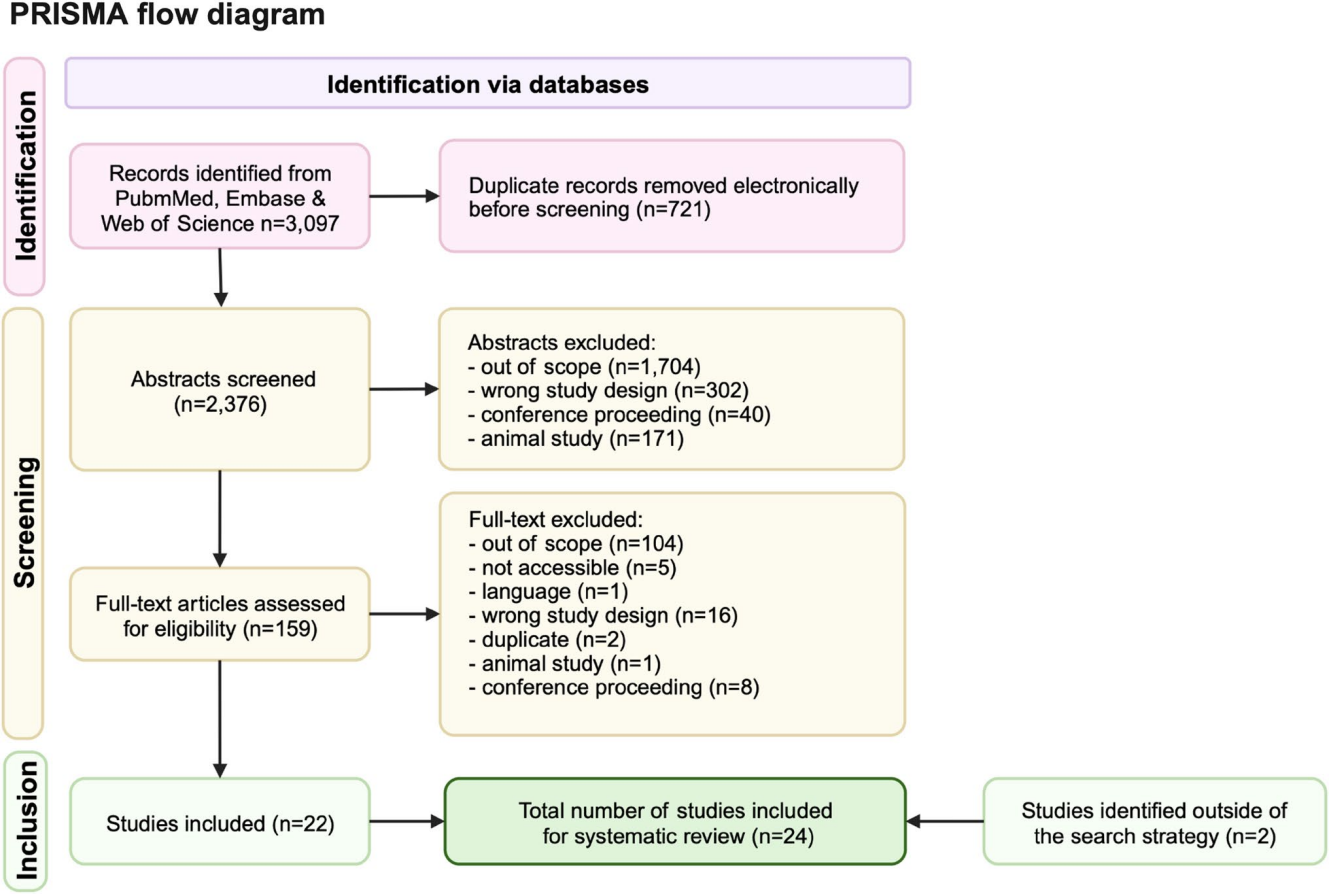
A PRISMA flow diagram outlining the exclusion/inclusion process

A systematic search was carried out on 23/12/2022 using the PubMed, Embase, and Web of Science databases. For the search, two search terms were used. Term 1 was ‘stroke’, ‘CVA’, or ‘cerebrovascular accident’ and Term 2 was ‘fundus photograph’, ‘fundus image’, ‘fundus autofluorescence’, ‘retinal image’, ‘retinal photograph’, ‘retinal autofluorescence’, ‘OCT’, ‘optical coherence tomography’, ‘OCTA’, ‘OCT A’, ‘adaptive optics’, ‘fluorescein angiography’, ‘optical imaging’, or ‘optical image’. Our search included human species and English language filters. On 21/09/2023, we repeated our search to ensure that this review was up to date. The search strategy is summarized in Fig. 2.

### Inclusion and exclusion criteria

Articles eligible to be included in this review were required to meet the following criteria:

1. The article discussed the role of retinal biomarkers as indicators of stroke risk.
2. The study was original and prospective or used data from biobanks involving prospective recruitment.
3. The study was conducted using human subjects.
4. The article was written in English.

Articles meeting the following criteria were excluded from our review:

1. Animal studies.
2. Non-original articles.
3. Case reports.
4. Conference abstracts.
5. Duplicates.
6. Preprint studies.
7. Articles which could not be obtained in full.
8. Studies which observed the relationship between *established* stroke and retinal biomarkers.
9. Studies observing the relationship between specific retinal diseases and stroke risk, unless providing individual biomarkers / retinopathy grades which can be ascertained using retinal imaging.

The reference lists of included articles were scanned for further articles which may fall within the scope of this review. All abstracts were screened by two authors independently (AS and ZG) who were blinded to each other's decisions. Conflicts were resolved by consensus between three authors (THJ, ZG, AS). All papers retained during abstract screening were screened in full by one author, with consensus discussions for any papers in which an inclusion/exclusion decision was not clear. Where a study was not available in an open access format, access was sought through three institutional libraries. Two recently published studies were identified outside of the search strategy and had come to the attention of the authors due to the high impact nature of the publications.

### Data collection

Data were extracted from each study included: population size; demographics; stroke subtype; ocular imaging used; software for image analysis used; retinal features; and compliance with STrengthening the Reporting of OBservational studies in Epidemiology (STROBE) and Advised Protocol for OCT Study Terminology and Elements (APOSTEL) reporting guidelines[12]. All included studies were reviewed in full by ≧2 authors prior to documentation of their findings.

### Synthesis of results

**Fig. 3:**
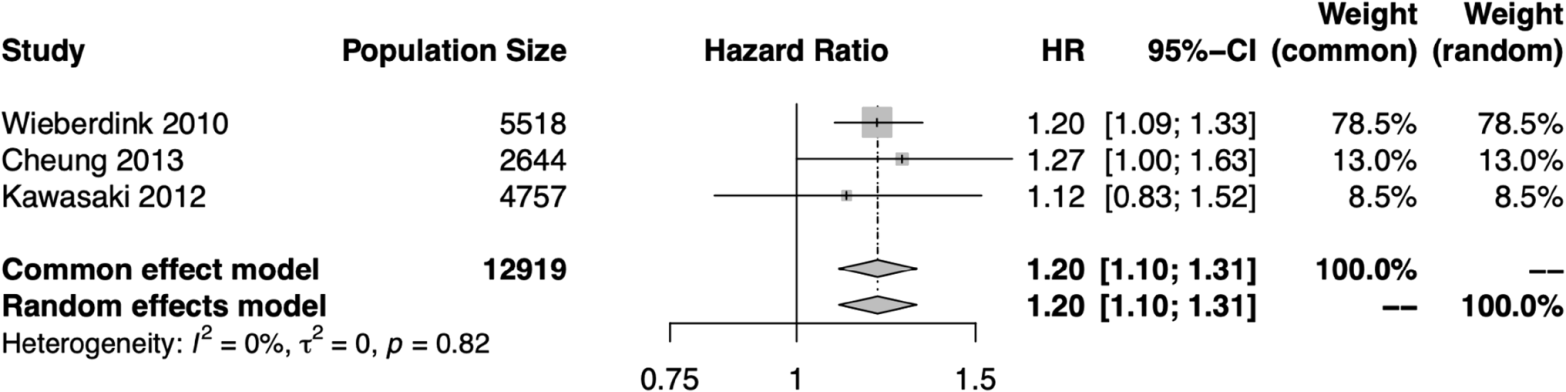
Hazard ratios (HR) for the central retinal vein equivalent (CRVE)–stroke relationship in each study eligible for meta-analysis, and the pooled HR and 95% confidence interval (CI) in both fixed effects and random effects models. There was no significant heterogeneity among these studies, and the results support a significant relationship between CRVE and stroke risk

**Fig. 4:**
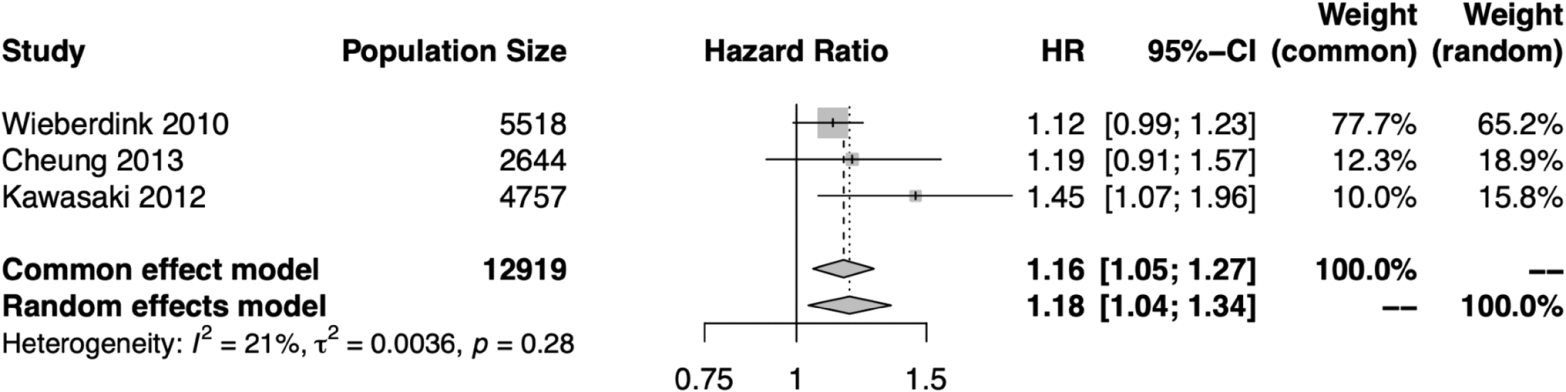
Hazard ratios (HR) for the central retinal artery equivalent (CRAE)–stroke relationship in each study eligible for meta-analysis, and the pooled HR and 95% confidence interval (CI) in both fixed effects and random effects models. There was not significant heterogeneity among these studies, and the results support a significant relationship between narrow CRAE and stroke risk

**Fig. 5:**
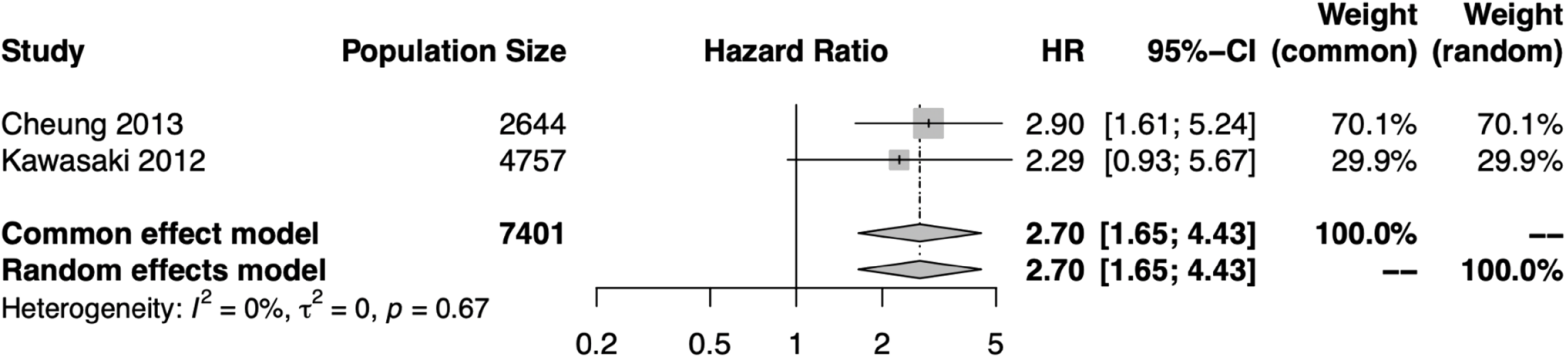
Hazard ratios (HR) for the presence of retinopathy–stroke relationship in each study eligible for meta-analysis, and the pooled HR and 95% confidence interval (CI) in both fixed effects and random effects models. The HR of retinopathy is compared to a reference of no retinopathy. There was not significant heterogeneity among these studies, and the results support a significant relationship between retinopathy and stroke risk

For most measures, meta-analysis was precluded by overlapping populations, heterogeneous retinal biomarker assessment, and heterogeneous populations. Where meta-analysis was performed, the calculations were conducted in R version 4.2.2. using the meta library version 6.5–0 [13]. The generic inverse variance method was used to produce pooled hazard ratios (HR), 95% confidence intervals (CI), and p values. Values reported in text are those derived from the random effects model although fixed effects estimates are also reported in figures. The restricted maximum-likelihood estimator for tau^2^ was used to identify heterogeneity. Forest plots are provided (Figs. 3, 4, 5). Our study is presented in accordance with the PRISMA guidelines.

### Assessment of bias

STROBE recommendations were used to assess the observational studies included in this review [12]. APOSTEL recommendations were used to assess the reporting of OCT results of studies included in this review [14].

### Compliance with ethical guidelines

This article is based upon previously published studies. There are no ethical concerns with regards to this study.

## Results

### Study Characteristics

The literature search heralded 3,097 results, of which 22 were eligible for inclusion. Two additional eligible studies were identified outside of the search strategy. Therefore, a total of 24 articles were included in this review (Figure 2). The included papers pooled hemorrhagic and ischemic stroke into a single outcome unless otherwise specified.

### Artificial intelligence (AI) enabled techniques to predict stroke

AI has enabled efficient analysis of enormous datasets, allowing for well-powered analyses; and the nature of models developed are becoming increasingly sophisticated in terms of the types of retinal variables being considered. In the domain of stroke prediction, there are currently three published AI-enabled prediction models, which illuminate the enormous potential for AI technologies to revolutionize this area of research.

The first of these is a methodologically robust study authored by Rudnicka et al., who utilized AI-enabled retinal vasculometry (developed using a mixture of supervised machine learning and deep learning) for the purpose of predicting stroke incidence and mortality [5]. In this study, the model was developed in a UK Biobank (UKBB) cohort (*n* = 88,052), and externally validated in the European Prospective Investigation into Cancer (EPIC) Norfolk cohort (*n* = 7,411). The external validation in this study is key and assessed if the models decisions are trustworthy and consistent across the diversity of datasets. QUARTZ was implemented as an automated solution for the processing of a substantial number of retinal images, enabling the extraction of quantitative vascular measurements [15]. The authors compared three models for the prediction of stroke and stroke mortality: 1) the Framingham risk score (FRS), 2) a combination of age, smoking status, medical history, and retinal vasculometry (RV), 3) a combination of FRS and RV. The addition of RV to the FRS model did not improve model performance, but the model combining age, smoking status, medical history, and RV showed similar performance to FRS with C-statistic of 0.73 in men and 0.75 in women (compared with 0.74 for both men and women with FRS). There was a comparable drop in performance during external validation across models. For circulatory mortality, the model inclusive of age, smoking, medical history, and RV performed well in men and women both in UKBB and EPIC Norfolk (C-statistics ranging from 0.75 to 0.77). This study demonstrates potential for AI-enabled retinal image analysis considering numerous retinal traits concurrently to be used for the prediction of stroke but does not outperform traditional risk scores.

Zhu et al. developed a deep learning-based model which estimated a subject’s age based upon their retinal photograph, and then utilized the ‘retinal age gap’ to assess risk of stroke [16]. In order to develop an algorithm to predict age, the authors initially trained a Xception deep learning model (a deep convolutional neural network architecture) [17] on UKBB subjects without any medical history (*n* = 11,052). Strong correlation was achieved between predicted age and chronological age in the healthy subject dataset (0.81, *p* <0.001). The study then assessed the role of the retinal age gap (defined as retina determined age minus true chronological age) in the prediction of future stroke in those who were stroke-free at baseline (*n* = 35,304). In a confounder adjusted model, each one-year increase in the retinal age gap was associated with a 4% increase in the risk of stroke (HR:1.04, 95% CI 1.00–1.08, *p* = 0.03). Furthermore, compared to participants with retinal age gap in the first quintile, participants with retinal age gap in the fifth quintile had significantly higher risks of stroke events (HR: 2.37, 95% CI 1.37–4.10, *p* = 0.002), whilst those in other quintiles did not differ significantly. The predictive performance for 10 year stroke risk assessed by the FRS was compared with the predictive value of a retinal age-based model adjusted for cardiovascular risk-related covariates. The AUCs of the retinal age-based model (0.68, 95% CI 0.64–0.71) were marginally higher than that of the FRS (0.66, 95% CI 0.63–0.69); however, this difference was not significant (*p* = 0.51). A notable weakness of this study was that the findings were not externally validated, and so it was unclear to what extent the model would generalize.

The most recently published study in this area was undertaken by Zhou et al. [18]. The study had a broad focus, using fundus photographs and OCTs for a range of detection and prediction tasks for both ocular and systemic diseases. The authors developed their foundation model, ‘RETFound’ (a masked autoencoder), using 904,170 color fundus photographs and 736,442 OCT images derived primarily from the Moorfields Diabetic imAge dataSet (MEH-MIDAS, *n* = 37,401, providing 90.2% of fundus photographs and 85.2% of OCTs). They utilized the RETFound encoder (from a masked autoencoder) and a multilayer perceptron for the three-year prediction of systemic disease, such as stroke, myocardial infarction, and heart failure. To test the performance of RETFound for the prediction of systemic disease, the authors utilized the data of subjects from the AlzEye study (*n* = 353,157, inclusive of *n* = 1,263 stroke subjects), with external validation in UKBB (*n* = 82,885, inclusive of 154 stroke subjects) [19, 20]. Although RETFound had impressive performance in some areas (such as detection of ocular disease), the accuracy of the model for the three-year prediction of stroke was limited during external validation, with an AUROC of 0.75 in AlzEye and 0.59 in UKBB when using fundus photographs and 0.75 in AlzEye and 0.56 in UKBB when using OCT. The strengths of the work are the utilization of a range of cohorts, inclusion of ethnically diverse subjects, the large sample size, use of external validation, and the breadth of tasks which RETFound can be utilized for. Key weaknesses include the relative geographic homogeneity of the cohorts (given they are entirely derived from the UK), the development of the autoencoder in principally diabetic cohort, and the limited performance for stroke prediction.

The above studies have illustrated the potential for AI-enabled retinal image analysis to be used for prediction of stroke. At present, these models do not offer a clear advantage over conventional stroke risk scores with regard to predictive performance, but prove that retinal image analysis has the scope to predict cerebrovascular events.

### Individual retinal features as indicators of future stroke risk

For the most part, the included studies observed the relationship between individual retinal traits and stroke risk, rather than considering several traits together within a predictive model. Whilst the future of retinal imaging enabled stroke prediction will be founded in more complex models considering numerous retinal characteristics, the following findings are interesting given that they prove the potential of the retina as an indicator of stroke risk, and demonstrate which retinal traits confer the most information about cerebrovascular health.

#### Vessel calibers and arterio-venous ratio

The calibers of retinal vessels are influenced by numerous systemic factors relevant to stroke, and reflect the health of the vasculature. As such, retinal arteriolar and venular calibers, and a measure of their relative diameters known as the arterio-venous ratio (AVR), have been explored as predictors of stroke. The venules are typically measured in the form of the ‘central retinal vein equivalent’ (CRVE), whilst the arterioles are measured in the form of the ‘central retinal artery equivalent’ (CRAE) [21].

Three studies were sufficiently similar to allow meta-analysis of the relationship between CRVE and CRAE and stroke risk (*n* = 12,919)[22–24]. Meta-analysis indicated that increasing CRVE (HR 1.20, 95% CI 1.10–1.31, *p* <0.0001) and narrower CRAE (HR: 1.18, 95% CI 1.04–1.34, *p* = 0.01) are significantly related to stroke risk (Figs. 3 and 4). Rather than using the CRVE or CRAE as measures of vascular caliber, Witt et al. (Beaver Dam Eye Study, BDES, *n* = 557) utilized an alternative image analysis pipeline which produced averaged vessel diameters [25]. In this analysis, narrower arteriolar diameter was of borderline significance in relation to stroke risk (*p* = 0.06), whilst AVR and venular diameter were not significant. It is important to note the smaller population size of this study (*n* = 28 stroke cases), which may account for the non-significant findings.

Wentzel et al. (Sympathetic Activity and Ambulatory Blood Pressure in Africans (SABPA) study) sought to compare the predictive power of retinal parameters between African (*n* = 139) and “Caucasian” (*n* = 178) subjects [26]. The study found that in those identified as African, wider venular calibers were predictive of an increased stroke risk; this association was not found in the “Caucasian” subgroup. Whilst it is feasible that there are true differences in the role of retinal parameters across ethnic groups, the population size of this study was small and the two ethnic groups were significantly different at baseline. Therefore, it appears unlikely that the difference between groups is truly underpinned by ethnicity.

Parameters linked to retinal vessel diameter have also been explored as predictors of stroke within exclusively diabetic cohorts. In a study of those with type 1 diabetes by Roy et al., (*n* = 725), no relationship between stroke and CRVE was found, and although narrower CRAE was significantly related to stroke risk over the six-year follow-up period in a univariate analysis, this did not persist after adjustment for confounders [27]. In a study of the Wisconsin Epidemiologic Study of Diabetic Retinopathy (WESDR) type 1 diabetic cohort by Klein et al., AVR was not significantly related to stroke risk over the course of a 20 year follow-up period (*P* = 0.15) [28]. Similarly, Sandoval–Garcia (*n* = 1,066, Edinburgh Type 2 Diabetes Study) failed to identify any significant relationship between CRVE, CRAE or AVR and combined stroke and TIA risk. Opposing these findings, a separate analysis in the WESDR cohort (*n* = 1,370) over a longer follow-up period of 22 years found that narrower CRAE and larger CRVE was associated with 22-year stroke mortality in those with type 2 diabetes (HR:1.47; 95% CI 1.04–2.07; *p* = 0.03 & HR:1.71; 95% CI 1.20–2.44; *p* = 0.003 respectively) when other stroke mortality-related risk factors were controlled [29].

Only one study has compared how strongly retinal vessel diameters are related to specific stroke subtypes (Rotterdam study, *n* = 5,518) [24]. In this analysis, larger CRVE was independently associated with an increased risk of both cerebral infarction (HR: 1.28; 95% CI 1.13 to 1.46), and intracerebral hemorrhage (HR: 1.53; 95% CI 1.09 to 2.15). CRAE, on the other hand, had a non-significant association with risk of cerebral infarction (HR: 1.12; 95% CI, 0.98 to 1.27), and intracerebral hemorrhage (HR: 1.25; 95% CI 0.87 to 1.79).

Finally, one study (*n* = 652) observed the relationship between retinal vessel diameter and recurrent stroke in those with established ischemic stroke. In this study, De Silva et al. found that there was no relationship between vessel calibers and risk of recurrent disease (CRAE *p* = 0.76; CRVE *p* = 0.94) [30].

In summary, the literature suggests that narrower retinal arterioles and wider venules are likely associated with increased risk of stroke and increased risk of stroke-related mortality. These findings appear to also apply to those with type 2 diabetes, but further study is required to assess the role of these markers in those with type 1 diabetes. There is little evidence that AVR predicts stroke on the basis of the limited literature available regarding this measure.

#### Retinal vessel tortuosity

Retinal vessel tortuosity is thought to reflect underlying vascular disease, and as such has been targeted as a measure of interest in the prediction of stroke. Cheung et al. (*n* = 3,189) identified that straighter retinal arterioles (HR: 0.38; 95% CI 0.15–0.94, comparing second versus first quartiles) are associated with risk of stroke, but this association did not remain significant when retinal arteriolar tortuosity was analyzed as a continuous variable. [22] Further, this study found that venular tortuosity measures had no relationship to stroke risk. Similarly, in the small study population analyzed by Witt et al., measures of retinal vessel tortuosity were not significantly related to stroke risk [31]. Contrastingly, in a study of individuals with type 2 diabetes (*n* = 1,066, Edinburgh Type 2 Diabetes Study), increased arteriolar tortuosity was found to have a significant relationship with stroke risk in models adjusted for cardiovascular risk factors and diabetic retinopathy (HR 1.31, 95% CI 1.01–1.68, *p* = 0.04) although this significance was attenuated when CRP was considered (*p* = 0.07) [32]. There was, however, no relationship between venular tortuosity and stroke risk in this study.

The literature surrounding the role of tortuosity as a predictor of stroke is therefore quite incongruous, and presently there is not strong evidence that tortuosity independently predicts stroke risk.

#### Retinal vessel complexity

Measures of retinal vascular complexity have been analyzed in numerous studies of cardiovascular and neurological disease on the understanding that complexity reflects the global integrity of the cerebral vasculature [33]. The most common measure utilized is ‘fractal dimension’, which is a quantitative measure used to summarize vessel branching complexity. A further measure of branching complexity is the branching coefficient, which quantifies bifurcation geometry.

Whilst Cheung et al. (*n* = 3,189) did not ascertain a significant association between risk of stroke and fractal dimension (*p* = 0.6), Liew et al. (*n* = 3,143) found that the reduced fractal dimension predicted stroke mortality risk after controlling for conventional cardiovascular risk factors (HR: 1.26, 95% CI 1.06–1.51, *p* = 0.01) and a small study by Kawasaki et al. (*n* = 285) identified a significant relationship between fractal dimension and stroke incidence (OR 1.39, 95% CI 1.06–1.83) [22, 34, 35]. Importantly, Liew et al. followed their patients over a longer duration of follow-up than Cheung et al. (12 years versus a median of 4.41 years), and although assessing mortality rather than incidence, appears to provide strong competing evidence against the findings of Cheung et al.

The association between fractal dimension and stroke has also been assessed in patients with diabetes. Sandoval-Garcia et al. (*n* = 1,066, Edinburgh Type 2 Diabetes Study) found that in those with diabetes, increasing fractal dimension was associated with decreased stroke risk in fully adjusted models, including when adjusting for CRP and diabetic retinopathy (HR: 0.7, 95% CI 0.51–0.94, *p* = 0.02) [32].

Finally, the importance of retinal vessel complexity has also been assessed in relation to stroke recurrence in those who have already suffered an ischemic stroke event (*n* = 196) [36]. In this work, it was found that higher bifurcation coefficient of arterioles is inversely related to stroke recurrence within one year of retinal photographs being obtained (OR: 0.36; 95% CI 0.20–0.64; *p* = .001).

Overall, the above evidence supports the assertion that increasing retinal vascular complexity is associated with decreasing risk of incident stroke and stroke-related mortality, and that this significance is independent of conventional cardiovascular risk factors.

#### Retinopathy

Numerous features of retinopathy have been assessed as predictors of stroke, including microaneurysms, hemorrhages, exudates, arteriovenous nicking (AVN), and focal arteriolar narrowing. These measures have been studied because they not only reflect specific disease processes (such as diabetes or hypertension), but also because they are indicative of underlying vascular disease.

There is broad agreement that retinopathy is an indicator of future stroke risk. Two studies observing the relationship between retinopathy of any type and stroke risk were eligible for meta-analysis (*n* = 7,401) [22, 23]. Analysis of these studies indicated that the presence of retinopathy is associated with increased risk of stroke (HR: 2.70, 95% CI 1.65–4.43, *p* < 0.0001, Fig. 5). Of these, one study performed an additional analysis excluding individuals with diabetes, finding that retinopathy in persons without diabetes was still associated with increased risk of stroke (HR: 3.07; 95% CI 1.17–8.09; *p* = 0.02) [23]. Within a study in the ARIC cohort (n=10,358), Wong et al. calculated the relative risk (RR) of stroke conferred by presence of various individual retinopathy traits adjusted for conventional cardiovascular risk factors [37]. In this analysis, the relative risk from any retinopathy was 2.58 (95% CI 1.59–4.20), from microaneurysms was 3.11 (95% CI 1.71–5.65), from soft exudates was 2.55 (95% CI 1.27–5.14), from blot hemorrhages was 2.55 (95% CI 1.27–5.14), from flame-shaped hemorrhages was 2.26 (95 CI 1.00–5.12) and from AVN was 1.60 (95% CI 1.03–2.47). Contrastingly, in a smaller study by Wong et al. (The Cardiovascular Health Study, *n* = 1,992), it was found that focal retinal signs (retinopathy, focal arteriolar narrowing, and AVN) were not associated with incident CHD or stroke [38]. Further, in the study by Wentzel et al. (SABPA, *n* = 317), it was found that whilst AVN was associated with stroke risk in African patients (OR 1.06, *p* =  0.05), this was not true in Caucasians, which again must be considered in the context of relatively small sample sizes and significantly different baseline characteristics [26].

The role of cerebral white matter lesions (WML) as an adjunct to retinopathy has also been explored (ARIC, *n* = 1,684) [39]. Within this work, it was found that relative to the absence of WML or retinopathy, the multivariable-adjusted RR for stroke was 2.6 (95% CI 0.9–7.6) for presence of WMLs only, 3.7 (95% CI 1.2–11.8) for retinopathy only, and 18.1 (95% CI 5.9–55.4) for both WMLs and retinopathy. These results may suggest that a combination of findings of retinopathy plus WML is a stronger indicator of future stroke risk than either of these markers in isolation.

Presence of hypertensive retinopathy and increasing severity of retinopathys have been shown to be associated with increased risk of long-term stroke in a study by Ong et al. within the ARIC cohort (*n* = 2,907) [40]. Specifically, it was found that after adjusting for conventional cardiovascular risk factors (including blood pressure), increasing severity of hypertensive retinopathy was associated with an increased risk of incident stroke, including cerebral infarction. Furthermore, even in medicated patients with good hypertension control, those with mild (HR: 1.96, 95% CI 1.09–3.55) and moderate hypertensive retinopathy (HR: 2.98, 95% CI 1.01–8.83) were at an increased risk of cerebral infarction.

Diabetic retinopathy has been shown to predict stroke. The study of patients with type 1 diabetes by Klein et al. (WESDR, *n* = 996) showed that the 20-year age-adjusted cumulative incidence for stroke was 5.9%, and presence and increasing severity of diabetic retinopathy was associated with stroke (OR: 1.6, 95% CI 1.1–2.3, *p* = 0.1) [28, 41]. A study within the ARIC cohort carried out by Cheung et al. (*n* = 1,617) showed similar findings, identifying that after adjusting for a comprehensive set of traditional cardiovascular risk factors diabetic retinopathy was associated with an increased risk of ischemic stroke (HR, 2.34, 95% CI, 1.13–4.86) [41]. Increased risks for ischemic stroke were also present for all individual retinopathy features among diabetic patients, but were statistically significant only for retinal microaneurysms.

Finally, two studies have explored retinopathy as a predictor of recurrent cerebrovascular disease. De Silva et al. (*n* = 652) observed the risk of recurrent cerebrovascular events in those who had already suffered ischemic stroke, finding that patients with severe AVN (HR 2.28, 95% CI 1.20–4.33) were more likely to have a subsequent cerebrovascular event compared with those without AVN[30]. They also showed that those with severe focal arteriolar narrowing were more likely to have a recurrent cerebrovascular event compared to those without narrowing (HR: 2.75, 95% CI 1.14–6.63). Klyscz et al. studied the relationship between retinopathy and recurrent cerebrovascular events in those with recent TIA or minor stroke (*n* = 722) [42]. In their study, a relatively simple, qualitative measure of retinopathy was applied, considering arteriolar narrowing, arteriovenous nicking, and rarefaction of vessels; and applying three grades of disease: no/mild vascular retinopathy, moderate vascular retinopathy, and vascular retinopathy with vessel rarefaction. Using this measure, no significant relationship was identified between moderate retinopathy (*p* = 0.28) or retinopathy with vessel rarefaction (*p* = 0.35) and recurrent stroke events, relative to those with no/mild retinopathy in a model adjusted for numerous confounders.

In summary, the literature supports the assertion that retinopathy is an indicator of higher stroke risk and suggests that specific individual features of retinopathy including AVN and microaneurysms may individually have predictive power.

#### Retinal vascular occlusions

Presence of retinal arteriolar emboli also suggests increased risk of stroke-related mortality. Wang et al. pooled data from the BDES (*n* = 4,926) and the BMES (*n* = 3,654) to show that there was increased stroke-related mortality independent of other cardiovascular risk factors in patients with presence of retinal arteriolar emboli (HR: 2.0, 95% CI 1.1–3.8). Furthermore, long-term cumulative stroke-related mortality was more than two-fold higher among participants with emboli than without emboli at baseline (HR: 2.5; CI, 1.4–4.4) [43]. Klein et al. studied the BDES cohort alone, and reported similar findings, identifying that those with retinal emboli had significantly higher hazard of stroke-related death (HR: 2.61, 95% CI 1.12–6.08) relative to those without retinal emboli [44]. Although outside the scope of this review in which we have focused on retinal features elicited in studies of retinal imaging findings, the findings for retinal emboli are of course in keeping with other acute retinal ischemic events. Most obviously, several studies have linked central retinal artery occlusion (CRAO) with stroke, both with those who have had CRAO being at risk of future stroke and vice versa [45–47].

### Risk of Bias

STROBE guidelines were used to assess the reporting of observational studies according to accepted standards, with the maximum possible score being 22 [12]. Reporting standards were generally high in papers included in this review, with STROBE scores ranging from 17 to 22 (Table 1). Reporting in studies utilizing OCT was assessed against the APOSTEL recommendations (Table 2) [14]. Whilst study compliance with APOSTEL recommendations was incomplete, the biobanks utilized have clearly protocolled methodologies.

**Table 1:**
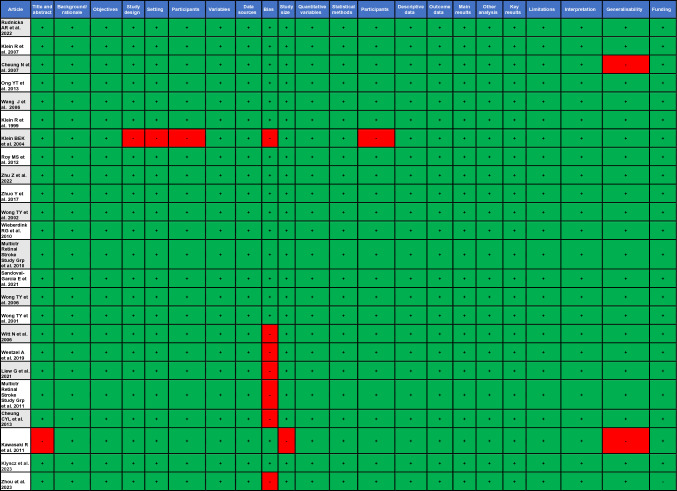
STROBE compliance checklist

Red non-compliant with this aspect of the checklist. Green compliant with this aspect of the checklist

**Table 2:**
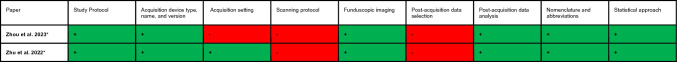
APOSTEL criteria compliance checklist

Red non-compliant with this aspect of the checklist. Green compliant with this aspect of the checklist

^*^Please note that studies above used publicly available biobanks to obtain OCT data and have referenced relevant sources for further details regarding image acquisition and analysis

## Conclusions

### The current state of evidence

Although the literature surrounding the role of retinal image derived biomarkers for the assessment of stroke risk has been developing for over two decades, this field in many ways remains in its infancy, with OCT and OCT-A biomarkers having received perishingly little attention. The published evidence illustrates that key individual retinal traits related to increasing stroke risk include wider CRVE, narrower CRAE, lower fractal dimension, increased arterial tortuosity, retinal emboli, and the presence of features of retinopathy. To a lesser extent, there is evidence that the presence of individual features of retinopathy, such as microaneurysms, AVN, and focal arteriolar narrowing, may predict stroke, although further studies in these areas are required given the conflicting evidence surrounding CRAE, and limited number of publications exploring individual retinopathy features.

The future of stroke prediction from retinal imaging will not be founded in the exploration of individual retinal features and their relationship to future stroke risk, but in construction of sophisticated models considering numerous retinal features. Early efforts toward this have been made in three studies which utilized sophisticated, AI-enabled analysis to demonstrate the opportunity for retinal images to be used to predict stroke [5, 16, 18]. Whilst current models do not perform sufficiently well to be rolled out into clinical practice, further work in this area could lead to cost-effective, quick, and accessible primary prevention programs based on retinal imaging.

### Opportunities and limitations

Although there is an enormous opportunity in this domain, there are numerous challenges which must be addressed before retinal imaging can be utilized to predict stroke in clinical practice.

First, the current models generally focus on selection of individual hypothesis-driven retinal parameters, rather than considering the retinal image in its entirety in a hypothesis free manner. Whilst this allows clear biological interpretability, it comes at the sacrifice of clinically useful data. It is understood that deep learning algorithms are capable of identifying clinically relevant patterns in images that cannot be appreciated by human investigators, and as such future research in this area should seek to analyze the full image using deep learning techniques, rather than isolating selected traits for consideration.

Second, OCT imaging is currently underexplored. Given the wealth of information conveyed by an OCT in terms of neuronal and vascular health, this is an area which is currently receiving insufficient attention. Particularly interesting imaging modalities include swept source OCT because of the improved visualization of the choroid and OCT-A which enables clear visualization of the microcirculation of the retina and choroid. Notably, no study included in this review explored the role of the choroidal vasculature in the prediction of stroke despite the recognized relationship between choroidal morphology and cardiovascular disease [48].

Careful external validation is essential for future papers modeling stroke prediction from retinal images. None of the AI-enabled predictive models evaluated in this review were tested in cohorts exclusively constituent patients with ocular conditions, such as cataract, age-related macular degeneration (AMD), or retinopathy. In the domain of MI prediction, it has been shown that the presence of AMD degrades predictive model performance, with decreasing performance with increasing grade of AMD [49]. This is a substantial problem, given that those at the highest risk of stroke are the elderly, who are also the most likely to suffer from the above ophthalmic diseases. Further, many studies identified in this review rely on cohorts which are not diverse with regard to age, ethnicity, or comorbidities, likely causing algorithmic bias in the developed models. This problem has been discussed at length in previous research, and it is essential that future research considers effective bias mitigation strategies and representative study design [50]. With this in mind, future work must evaluate how well models perform across diverse patient subgroups.

Finally, the included studies typically combined ischemic and hemorrhagic stroke into a single outcome. Whilst this increases power, it is likely that the degree to which retinal biomarkers predict different forms of stroke will vary, and as such it would be interesting for future research to aim to predict distinct stroke phenotypes. Most importantly, future studies should segregate stroke events into hemorrhagic and ischemic groups and observe the differences with regard to the accuracy of retinal imaging-informed predictions, and the specific retinal phenotypes of relevance to the risk of stroke events.

In conclusion, our review highlights early work that has revealed potential for retinal imaging to be used to predict stroke. In this review, we have highlighted the current evidence suggesting retinal features are linked to future stroke risk, limitations of this work, and opportunities for future researchers​​ to build on the existing evidence to produce models which are suitable for clinical practice. Although the currently available models for stroke prediction are unready for implementation in clinical practice, there is clear scope for clinical utility once the aforementioned limitations have been addressed. With development, a future in which your annual ophthalmic/optometric appointment could serve to analyze your cerebrovascular health in addition to your ocular health is within sight.

### Supplementary Information

Below is the link to the electronic supplementary material.

Supplementary file1. A web table containing key study details and a brief summary of the salient findings is provided (supplementary table 1.1). A description of retinal measures mentioned in this review, and the software use to perform the analyses detailed is provided in supplementary table 1.2. (XLSX 148 KB)

## Data Availability

Not applicable.
